# Imide-linked phthalocyanine-based covalent organic framework for electrochemical sensing of human epidermal growth factor receptor 2

**DOI:** 10.1039/d6ra04581b

**Published:** 2026-07-07

**Authors:** Lunathi Ncwane, Philani Mashazi, Tebello Nyokong

**Affiliations:** a Institute for Nanotechnology Innovation, Rhodes University P. O. Box 94 Makhanda South Africa t.nyokong@ru.ac.za +27 46 6038260

## Abstract

In this work, we present the design of a novel aptasensor based on an imide-linked, phthalocyanine-derived covalent organic framework (Im-COF) for the electrochemical detection of human epidermal growth factor receptor 2 (HER2). The electrocatalyst (cobalt phthalocyanine-based Im-COF) employed is a two-dimensional (2D) layered material with strong π–π interactions and push–pull electronic effects that enhance signal generation. To the best of our knowledge, Im-COF is applied for the first time in HER2 electrochemical detection. HER2 detection was performed using differential pulse voltammetry (DPV) over the concentration of 2.0–40 pg mL^−1^, enabling assessment of detection limits, selectivity, stability, repeatability and real-sample performance. Among the fabricated sensors, the GCE/Im-COF/Apt (Apt = aptamer) platform exhibited the lowest limit of detection (0.093 pg mL^−1^) and the highest analytical sensitivity. Furthermore, the fabricated biosensor (GCE/Im-COF/Apt) further showed good stability, repeatability, selectivity and favourable applicability for real-sample analysis.

## Abstract 2

In this work, we present the design of a novel aptasensor based on an imide-linked, phthalocyanine-derived covalent organic framework (Im-COF) for the electrochemical detection of human epidermal growth factor receptor 2 (HER2).

## Graphical abstract

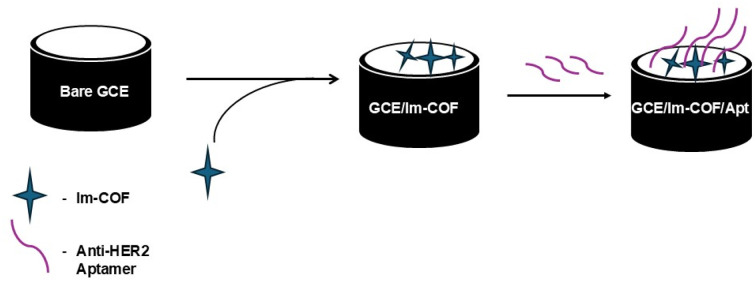

## 1. Introduction

There is a growing need for analytical methods that enable sensitive, and cost-effective detection of breast cancer biomarkers.^1–4^ Early and accurate biomarker quantification is essential for improving diagnostic outcomes and informing personalized treatment strategies.

In this work, we focus on the electrochemical detection of human epidermal growth factor 2 (HER2), a clinically important biomarker that is frequently overexpressed in aggressive breast cancer subtypes.^5^ Conventional HER2 detection methods, such as fluorescence *in situ* hybridization (FISH) and immunohistochemistry (IHC) are well-established but suffer from limitations including high cost, long processing times and operational complexity.^6–8^ These constraints have motivated substantial interest in electrochemical biosensors as promising alternatives, owing to their simplicity, high sensitivity, and suitability for point-of-care applications.^9–11^

Electrode materials play a crucial role in determining biosensor performance. Phthalocyanines (Pcs) represent a widely studied class of electrocatalysts because of their excellent electrochemical activity and structural tunability.^12–15^ Their ability to form extended π-conjugated systems enables integration into covalent organic frameworks (COFs), which provide high surface area, structural order, and chemical stability for sensing applications.^15,16^ Covalent organic frameworks (COFs) are porous and crystalline two- or three-dimensional (2D or 3D) polymers made up of strong covalent bonds.^17^

Studies on COF based electrochemical biosensors for HER2 have been reported before.^17,18^ Among the diverse linkages for COF formation, imide linkages have attracted increasing attention due to their chemical and thermal robustness.^19^ Although phthalocyanine-based COFs have previously been explored for applications in electrocatalysis and nonlinear optics,^20–22^ this study reports their first use in HER2 detection, marking a significant advancement in the application of phthalocyanine based COF materials towards clinically relevant biomarker detection. The novelty of our approach lies in leveraging both the push–pull electronic affects of the organic linker and the strong π–π interactions inherent to the 2D layered architecture of Im-COFs (Im = imide), which together enhance electrochemical performance. While the aptamer used in this work is amino-terminated, immobilization was intentionally achieved through physisorption to simplify fabrication without compromising sensor functionality.

## 2. Experimental

Materials, equipment and synthesis are described in the SI.

### 2.1 Synthesis of imide linked covalent organic frameworks (Im-COF)

**Scheme 1:**
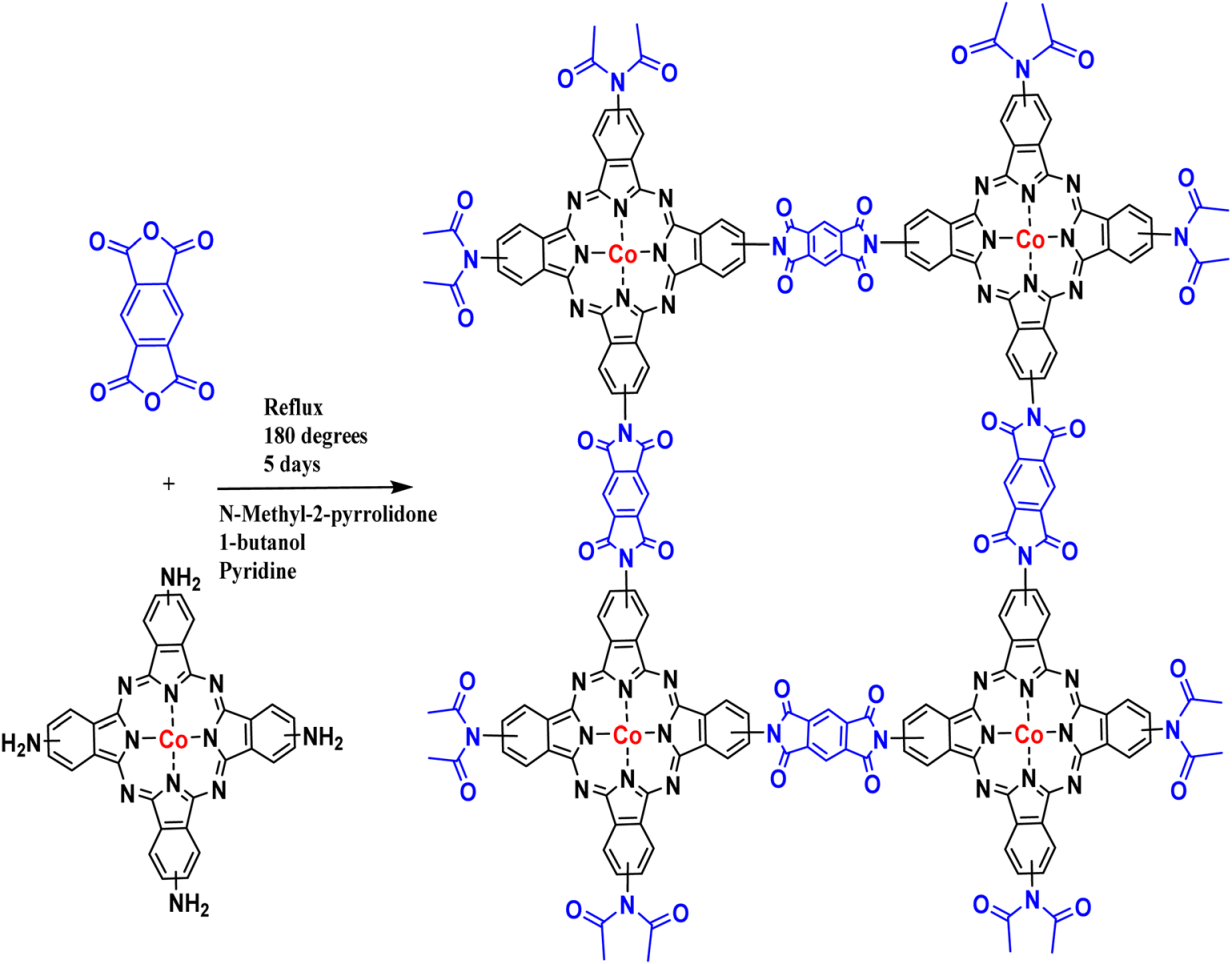
Imide linkage synthesis of Im-COF.

Cobalt tetraamino phthalocyanine (CoTAPc) was synthesized according to previously reported procedures.^23^ The imide-linked covalent organic framework (Im-COF) was prepared following literature methods with slight modifications,^24^ as illustrated in Scheme 1. Briefly, CoTAPc (40 mg 0.037 mmol) and pyromellitic dianhydride (22 mg 0.164 mmol) were added to a mixture containing *N*-methylpyrrolidone (NMP) (10 mL), *n*-butanol (10 mL), and pyridine (1 mL) in a round-bottom flask. The mixture was refluxed for at 180 °C for five days, after which it was cooled to room temperature. The resulting precipitate was thoroughly washed with dimethylformamide (DMF) and acetone to remove unreacted precursors and solvent residues.

### 2.2 Electrode modification

The glassy carbon electrode (GCE) (geometric area of 0.071 cm^2^) was used as a working electrode, platinum wire as a counter electrode and silver|silver chloride (3 M KCl) as the reference electrode. Prior to modification, the GCE was polished with aluminium oxide using Metrohm polishing pad and left to dry in air. The electrodes were modified by drop dry method as follows: the freshly cleaned GCE was modified by drop-casting 10 µL (1 mg mL^−1^ in DMSO) of CoTAPc or Im-COF. A –NH_2_ terminated aptamer (Apt, 10 µL) was sequentially immobilized onto the modified electrode surfaces *via* physisorption, forming GCE/CoTAPc/Apt and GCE/Im-COF/Apt. This non-covalent immobilization approach was selected to simplify fabrication while preserving sensor functionality.

### 2.3 HER2 detection

HER2 quantification was performed using differential pulse voltammetry (DPV). The modified electrodes were incubated for 2 h at room temperature in phosphate-buffer saline (PBS, pH 7.4) containing HER2 at concentrations between 2 and 40 pg mL^−1^. Electrochemical responses were recorded in [Fe(CN)_6_]^3−/4−^ (1 mM in 0.1 M KCl) in 10 mM PBS. Measurements were conducted at a scan rate of 10 mV s^−1^ over a potential window of 0–1 V at room temperature.

## 3. Results and discussion

Scheme 1 shows the synthesis of imide linked (Im-COF) from cobalt(ii) tetra amino phthalocyanine (CoTAPc) and pyromellitic dianhydride.^24^ The synthesized materials were characterized by various techniques such as ultra-violet visible spectroscopy, thermogravimetric analysis, X-ray diffraction, Fourier transform infrared spectroscopy. Time-of-flight secondary ion mass spectrometry (TOF-SIMS) was used for the determination of mass spectrum of the precursor, CoTAPc, Fig. S1 in SI.

### 3.1 Characterization of CoTAPc and Im-COF

Mass spectrum displayed in Fig. S1 (SI) shows successful synthesis of CoTAPc with calculated mass of 631.13*m*/*z* corresponding to obtained values 631.25*m*/*z*.

**Fig. 1:**
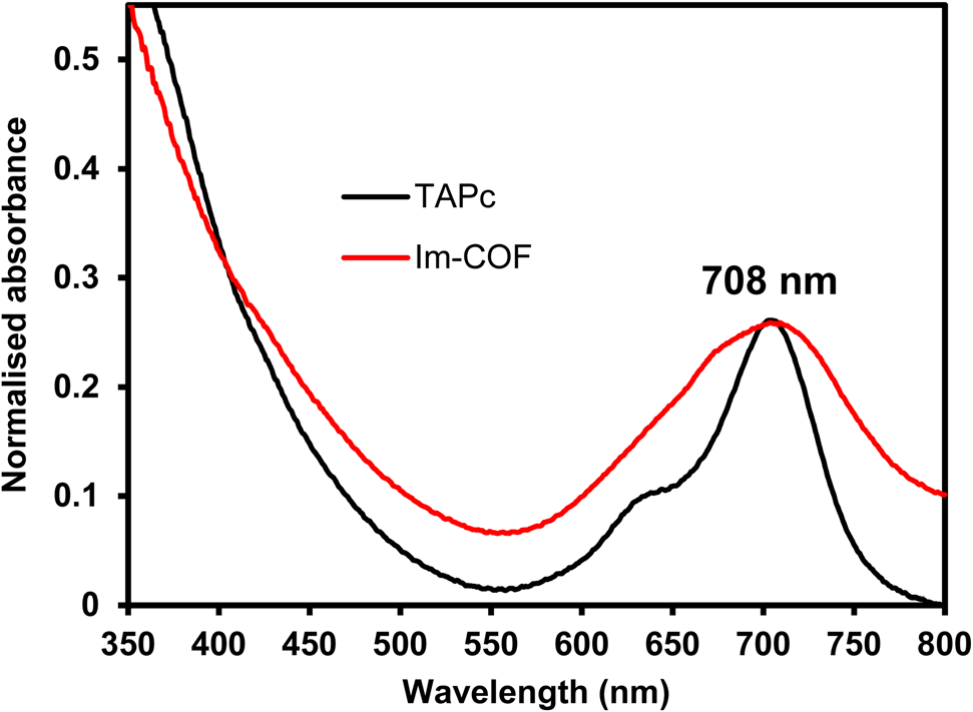
UV-visible spectra of CoTAPc and Im-COF.

UV-visible spectroscopy provided further structural validation, Fig. 1. Both CoTAPc and the imide-linked covalent organic framework (Im-COF) exhibited the characteristic Q-bands expected for phthalocyanine related materials,^25,26^ while notably broader Q-band observed for Im-COF indicates enhanced aggregation. The aggregation is attributed to the strong ππ interactions intrinsic to 2D conjugated COF architectures as well as the influence of extended metal centers within the framework.^22^

Fourier-transform infrared spectroscopy (FTIR) was employed to confirm the formation of imide linkages in the COF. The emergence of a prominent C–N–C stretching vibration at 1364 cm^−1^ and the disappearance of the –NH_2_ peak between 3300–3100 cm^−1^ as shown in Fig. S2, provide evidence of successful imide linkage between CoTAPc and pyromellitic dianhydride. The appearance of carbonyl (C=O) stretching bands at 1707 cm^−1^ and 1770 cm^−1^ for Im-COF, along with the absence of the C–O–C asymmetric stretch at 1240 cm^−1^ characteristic of the dianhydride precursor, further substantiates the formation of fully imidized framework.

**Fig. 2:**
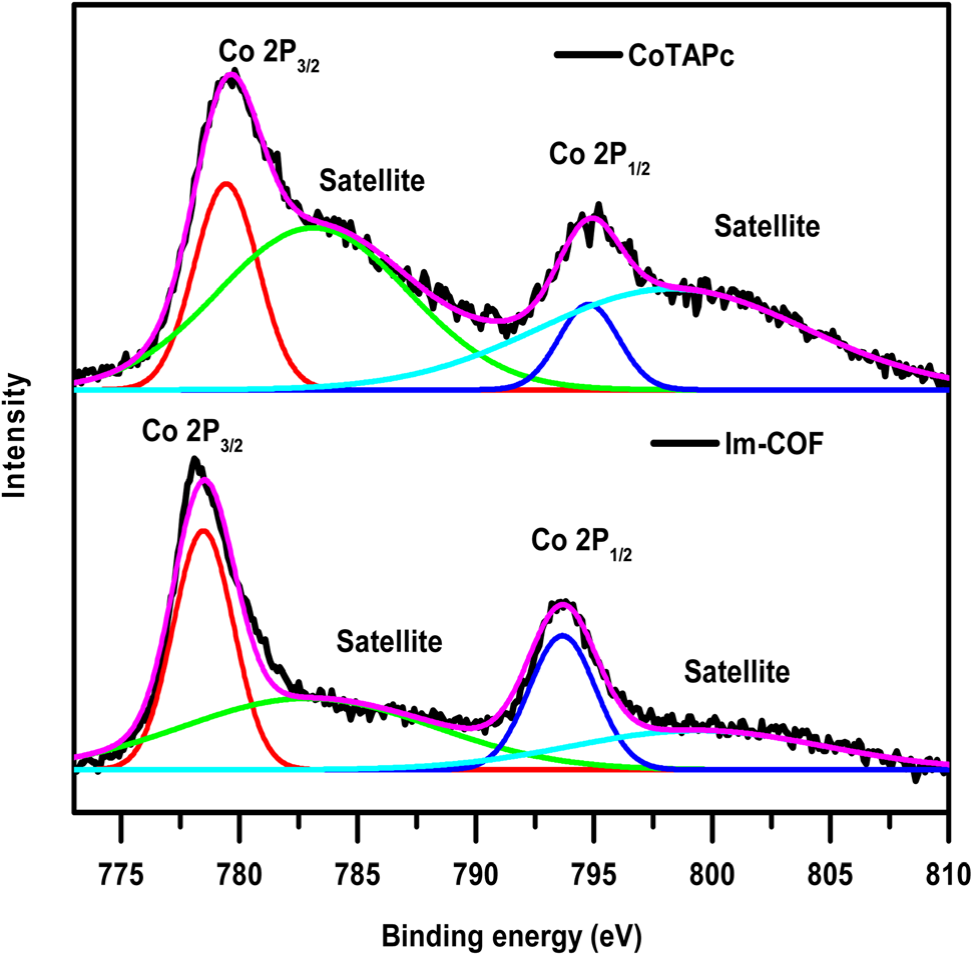
High resolution (Co 2p) X-ray photoelectron spectra of CoTAPc and Im-COF.

To further characterize the elemental composition and imide bond formation, X-ray photoelectron spectroscopy (XPS) analysis was conducted. The survey scan illustrated in Fig. S3a exhibited the characteristic peaks of an imide-linked Im-COF such as C 1s, N 1s, O 1s and Co 2p. The high resolution Co 2p spectra (Fig. 2) show the oxidation state of Co, where the presence of Co 2p_3/2_, Co 2p_1/2_ and satellite peaks correspond to Co^2+^ species of the phthalocyanine ring.^27^ CoTAPc showed Co 2p_3/2_ (779.5 eV) and Co 2p_1/2_ (795.2 eV) peaks, whereas Im-COF peaks shifted to 778.2 eV and 793.3 eV, respectively, which is attributed to increased electron density at the COF's cobalt centers due to the extended π conjugated system of the Im-COF.^28^ The high resolution N 1s spectrum (Fig. S3b) produced peaks at 398.5 eV, 399.4 eV and 401.5 eV corresponding to N–C/C=N, Co–N and N–(C=O)_2_ bonds, respectively. Furthermore, the C 1s spectrum (Fig. S3c) directly proves the C=O presence at 288.1 eV supported by the high-resolution O 1s spectrum (Fig. S3d), which showed a C=O peak at 529.5 eV. Overall, XPS analysis confirmed the successful preparation of the imide-linked COF.^28,29^

**Fig. 3:**
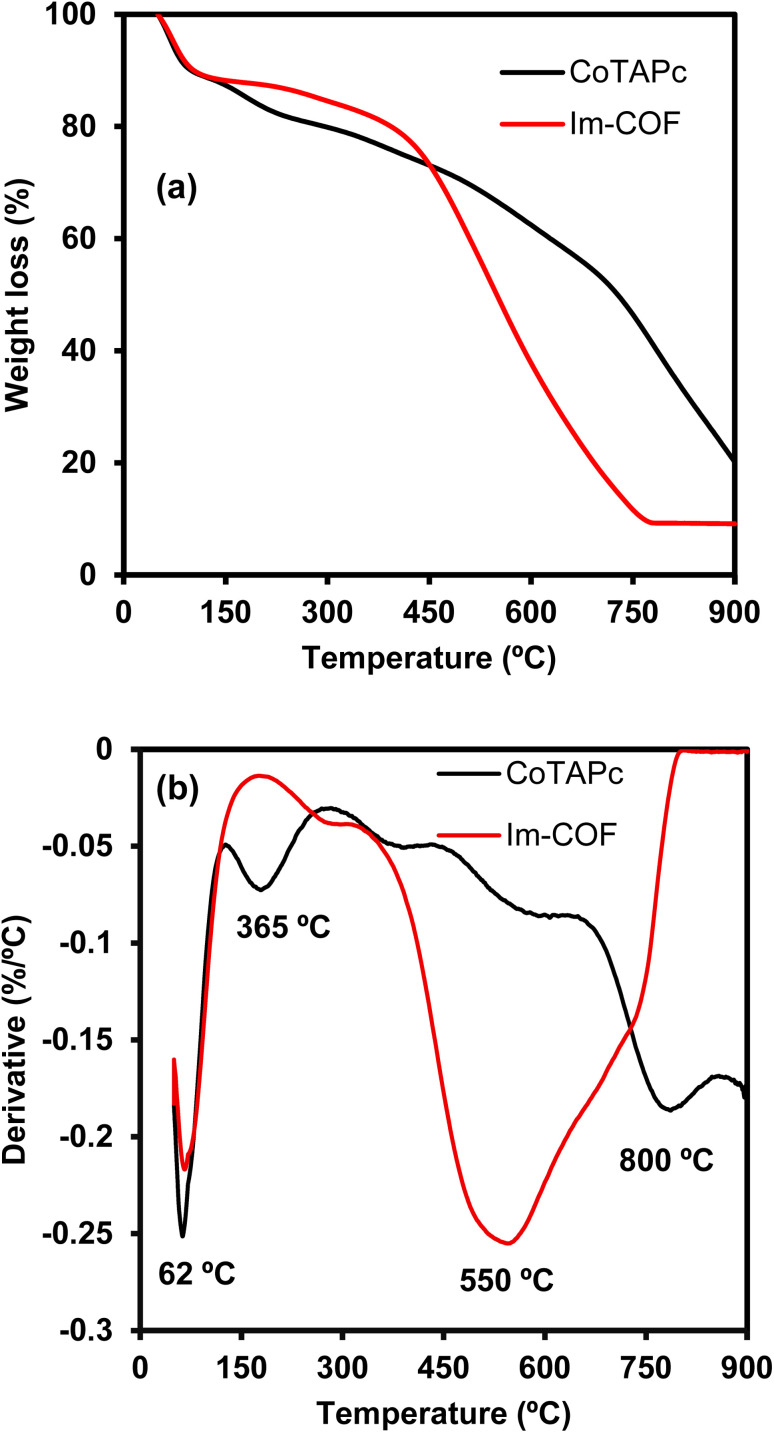
(a) Thermogravimetric analysis and (b) derivative TGA for the synthesized CoTAPc and Im-COF.

Thermogravimetric analysis (TGA) revealed distinct differences in thermal stability between the synthesized materials, Fig. 3. Both CoTAPc and Im-COF displayed initial mass loss around 62 °C due to desorption of water and residual solvents. However, Im-COF exhibited significantly higher thermal robustness, with major decomposition (isoindoline decomposition) occurring at 550 °C compared to 365 °C for CoTAPc, Fig. 3b. The enhanced stability is attributed to the strong ππ interaction and the presence of imide linkages connecting phthalocyanine monomers within the COF network. The thermal decomposition stages were further supported by derivative TGA displayed in Fig. 3b.

**Fig. 4:**
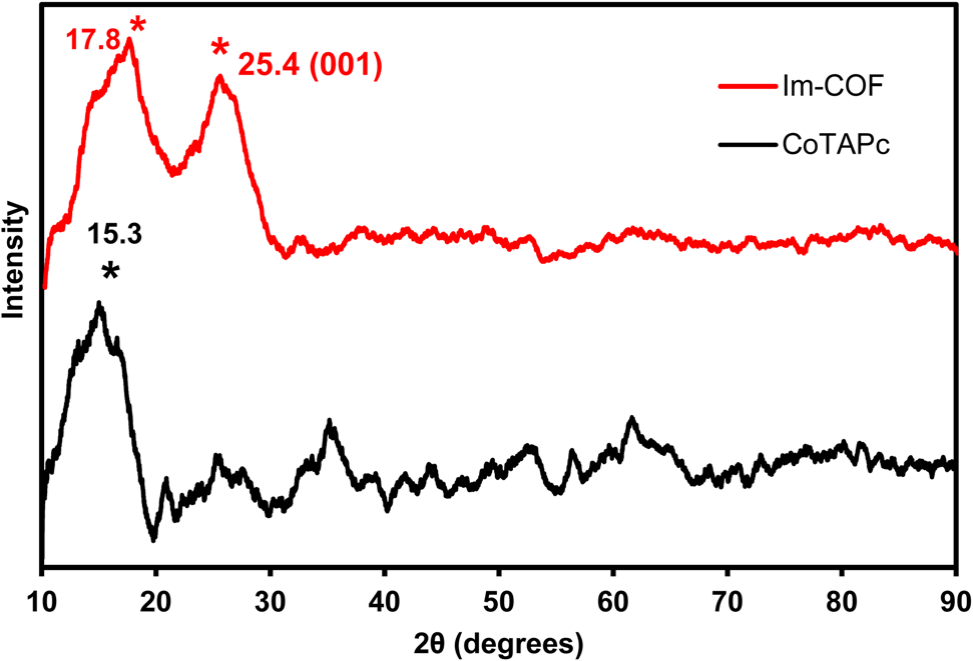
XRD patterns for CoTAPc and Im-COF.

COFs show both amorphous and semicrystalline properties.^30,31^ The type and level of crystallinity is mainly affected by reaction reagents and conditions. Fig. 4 displays X-ray powder diffraction (XRD) patterns of the synthesized materials. Both materials show semi-crystalline properties where Im-COF has an extra peak at 25.4° assigned to (001) facet mainly in ππ stacked 2D layered materials.^32–34^ The appearance and shift of peaks confirm COF formation.

**Fig. 5:**
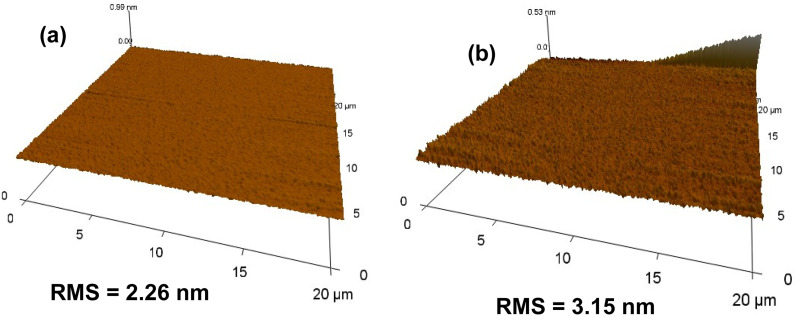
Atomic force microscope 3D surface topography images for (a) CoTAPc and (b) Im-COF.

The successful formation of 2D layered phthalocyanine-based COF (Im-COF) was confirmed by characterizing differences in surface topology. Atomic force microscopy (AFM) 3D images were recorded as illustrated in Fig. 5. The obtained 3D images exhibited a smooth surface topography with a roughness of 2.26 nm for CoTAPc, whereas Im-COF showed slightly increased roughness, with surface roughness of 3.15 nm. The 3D topography image and slight increase in surface roughness is attributed to the semicrystalline nature of a uniform 2D Im-COF film, which leads to disordered stacking.^35^ This is also evidenced by the XRD patterns in Fig. 4.

### 3.2 Electrode modification and characterisation

**Scheme 2:**
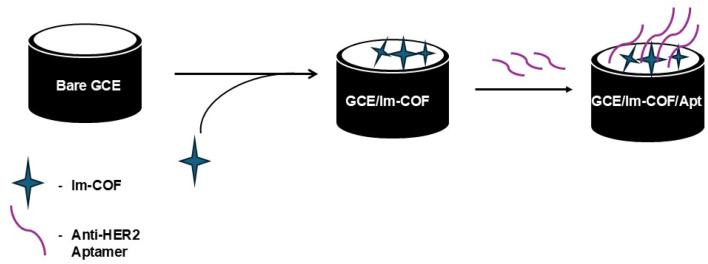
Bare glassy carbon electrode (bare GCE) drop-dry modification with Im-COF and anti-HER2 aptamer immobilisation.

Electrode modification was carried out using drop dry method and physisorption as illustrated in Scheme 2.

#### 3.2.1 Cyclic voltammetry in ferri/ferrocyanide

**Fig. 6:**
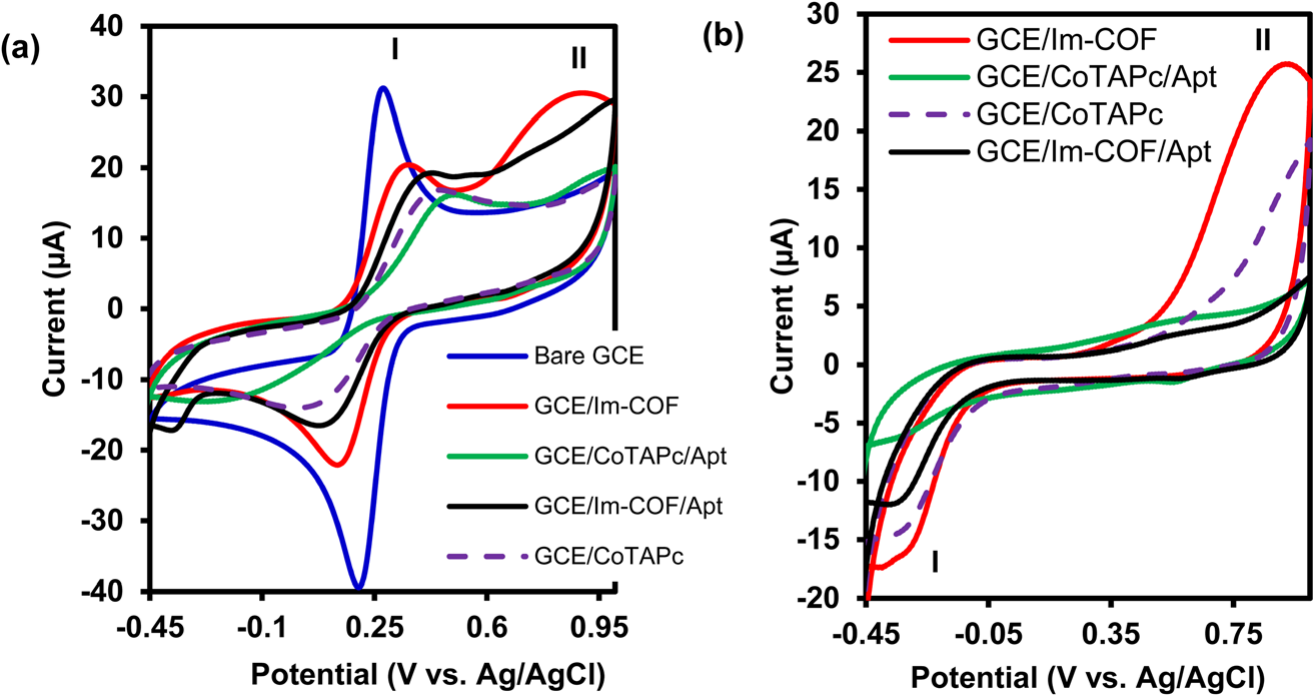
Cyclic voltammograms of Bare GCE, GCE/CoTAPc, GCE/Im-COF, GCE/CoTAPc/Apt and GCE/Im-COF/Apt (a) 1 mM [FeCN_6_]^3−/4−^ (in 0.1 M KCl) in 10 mM PBS (b) 10 mM PBS at 10 mV s^−1^. Apt = aptamer.

Electrode characterization was performed using cyclic voltammetry (CV) in 1 mM [FeCN_6_]^3−/4−^ (in 0.1 M KCl) in 10 mM PBS. The experiments are conducted to investigate electron transfer properties of the modified electrodes. CV curves are shown in Fig. 6a where GCE/Im-COF shows two peaks. Redox peak (I) is due to ferri/ferrocyanide redox process, while peak (II) is due to Co(ii)/Co(iii) redox processes.^36,37^ The Co(ii)/Co(iii) peak only appears for GCE/Im-COF due to the multiple linked CoTAPc centres species on the COF. The Co(ii)/Co(iii) redox peak for CoTAPc is not clearly pronounced, it may be masked by ferri/ferrocyanide processes.^12,38^ Electron transfer properties are determined using anodic-to-cathodic peak potential difference (Δ*E*_p_). Lower Δ*E*_p_ values indicate excellent electron transfer properties. The increase in Δ*E*_p_ results from insulated GCE surface. Aptamer immobilisation shows a significant electrode blockade, hindering electron transfer. Aptamers are known to be electrochemically inactive, hence the noticeable increase in Δ*E*_p_ (Fig. 6a and Table 1). Table 1 shows improved electron transfer properties (lower Δ*E*_p_) on GCE/Im-COF compared to GCE/CoTAPc in the presence and absence of the aptamer.

**Table 1:** Summary of Δ*E*_p_, *R*_ct_, Chi-squared (*χ*^2^) and surface coverage (*Γ*) values obtained from cyclic voltammetry and electrochemical impedance spectroscopy in [Fe(CN)_6_]^3−/4−^ (1 mM) in 0.1 M KCl and 10 mM PBS (pH 7.4)

Electrodes	Δ*E*_p_ (mV) in 1 mM [Fe(CN)_6_]^3−/4−^	*R*_ct_ (kΩ)	(*χ*^2^)	*Γ* (mol cm^−2^)
Bare GCE	73	0.22	0.019	—
GCE/CoTAPc	381	16.5	0.078	1.92 × 10^−7^
GCE/CoTAPc/Apt	496	39.0	0.340	8.42 × 10^−8^
GCE/Im-COF	197	3.81	0.050	2.76 × 10^−7^
GCE/Im-COF/Apt	305	12.4	0.191	1.77 × 10^−7^

#### 3.2.2 Electrochemical impedance spectrometry (EIS)

**Fig. 7:**
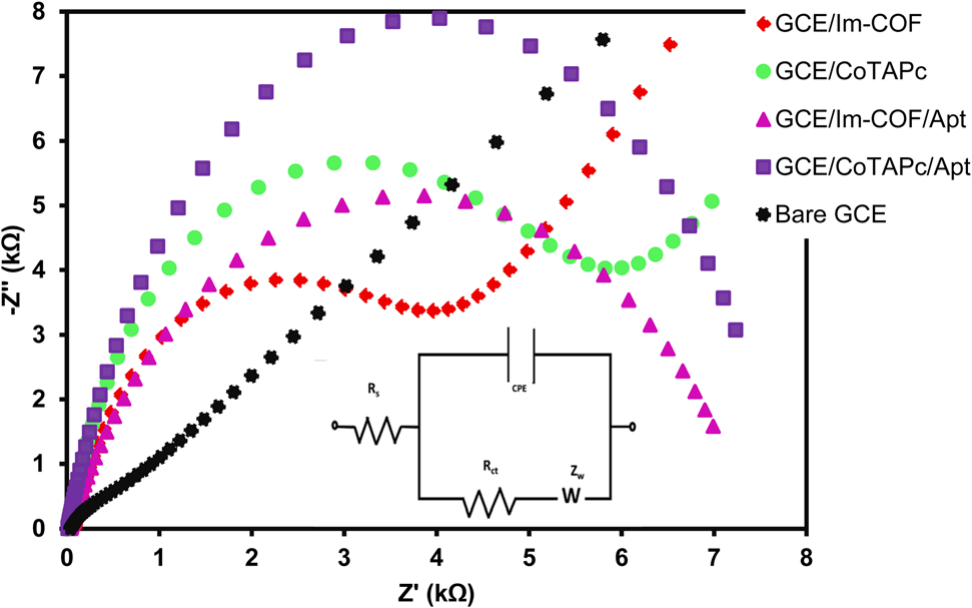
Comparative Nyquist plots in 1 mM [Fe(CN)_6_]^3−/4−^ (in 0.1 M KCl) in 10 mM PBS (pH 7.4). Inset: Randles fitting model employed.

Electrochemical impedance spectroscopy (EIS) is employed in this work to investigate charge transfer resistance of the designed aptasensors. Charge transfer resistance (*R*_ct_) is obtained from the fitting of Nyquist plot semi-circles using Randles circuit, Fig. 7.^39^Table 1 shows a summary of *R*_ct_ values, where GCE/Im-COF and GCE/Im-COF/Apt show the lower *R*_ct_ values compared GCE/CoTAPc and GCE/CoTAPc–Apt, respectively. The modification of bare GCEs results in insulating films hence the increase in *R*_ct_. *R*_ct_ values have inverse proportional relationship with conductivity and electron transfer. Lower *R*_ct_ values indicate good conductivity and electron transfer. In this case, the electrodes are further modified with anti-HER2 aptamer for specific HER2 bio-recognition. Aptamers are known for forming insulating films, hence there is an increase in *R*_ct_ values for both GCE/Im-COF/Apt (12.4 kΩ) and GCE/CoTAPc/Apt (39.0 kΩ) compared to GCE/CoTAPc (16.5 kΩ) and GCE/Im-COF (3.81 kΩ) without the aptamer. Aptamer immobilisation on modified electrodes results in electrostatic repulsion with the negatively charged [Fe(CN)_6_]^3−/4−^ due to negatively charged phosphate backbone of the aptamer, hence higher *R*_ct_ values and Warburg element disappearance in Fig. 7. The disappearance of the Warburg region indicates restricted diffusion controlled redox process by steric hindrance from negatively charged aptamer.

Chi-squared values were also obtained from the fitting results to measure the fitting errors as summarized in Table 1. All the obtained values were low indicating good fitting model.

The results shown in Table 1 are in agreement with Δ*E*_p_ from cyclic voltammetry experiments. The major improvements on conductivity for GCE/Im-COF are due to the availability of more Co^2+^ species, donor–acceptor electron transfer and strong ππ interactions as supported by cyclic voltammetry experiments.

#### 3.2.3 Surface coverage

Surface coverage of the modified electrodes was investigated by chronocolorimetric studies in [Fe(CN)_6_]^3−/4−^ (1 mM) in 0.1 M KCl and 10 mM PBS (pH 7.4). The potential was stepped from 0 V to 1 V for oxidation of Fe(CN)_6_^3−^ (aq) to Fe(CN)_6_^4−^ (aq). Firstly, effective surface area is calculated from integrated Cottrell equation (eqn (1)) and chronocolorimetric plots (Fig. S4).^40^1
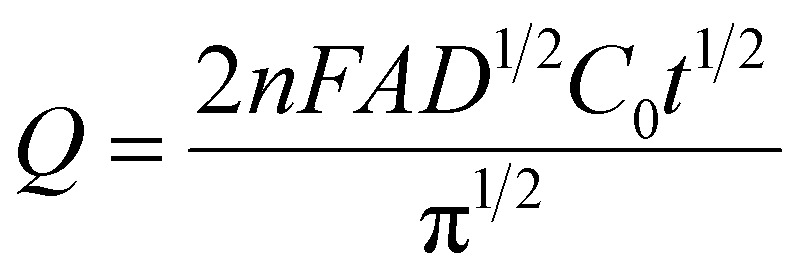
where *n* = number of electrons transferred, *A* = the effective surface area, *C*_0_ = concentration of [Fe(CN)_6_]^3−/4−^, *F* the Faraday's constant (96 485 C mol^−1^), *t* = time, *D* = diffusion coefficient (7.6 × 10^−6^ cm^2^ s^−1^) of [Fe(CN)_6_]^3−/4−^ according to literature.^41^

To determine the surface coverage, effective surface area obtained from eqn (1) and (2) are employed.^42^2
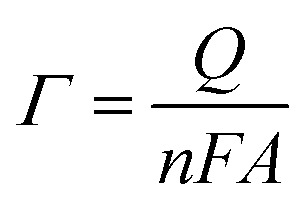
where *Γ* is the surface coverage, *Q* is the integrated surface area of the modified electrode, *n* the number of electrons transferred, *F* the Faraday's constant (96 485 C mol^−1^) and *A* is the effective surface area calculated from (eqn (1)). The integrated surface area (*Q*) was estimated by the integration of peaks (I) from Fig. 6b.

Table 1 summarises the calculated surface coverages from eqn (2). As shows in Table 1, Im-COF containing electrodes have higher surface coverages when compared to the corresponding CoTAPc. The obtained values were all higher than 1 × 10^−10^ mol cm^−2^ for monolayer coverage, revealing multilayer film formation.^43^ Higher surface coverages on GCE/Im-COF further shows extended π electron system and availability of more exposed electroactive sites (Co^2+^). The immobilisation of anti-HER2 aptamer on electrode surface decreased the surface coverage due to electrochemically inactiveness of aptamers.

### 3.3 HER2 detection (differential pulse voltammetry, DPV)

**Fig. 8:**
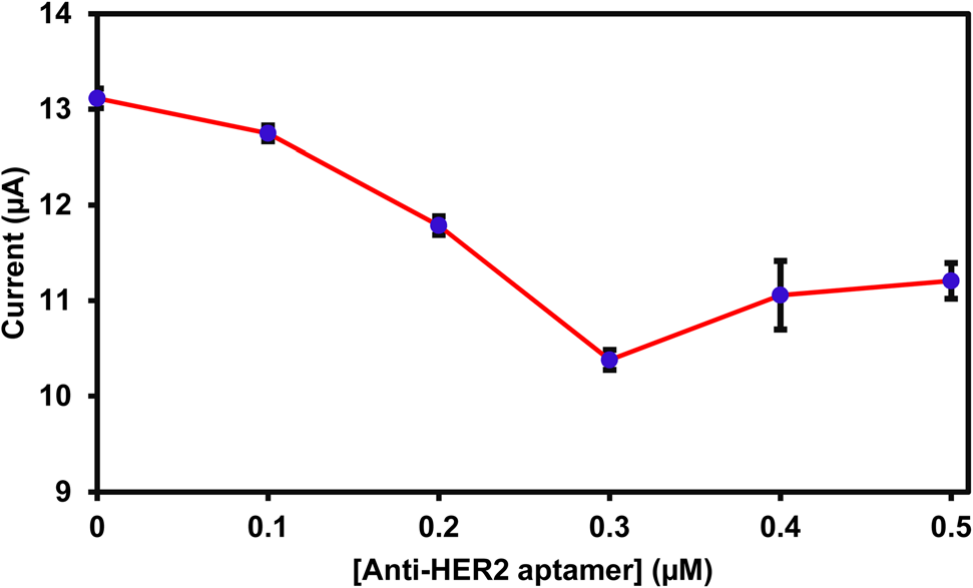
Plot of aptamer concentration *versus* current for GCE/Im-COF/Apt in 1 mM [Fe(CN)_6_]^3−/4−^ (in 0.1 M KCl) in 10 mM PBS (pH 7.4).

Firstly, optimization experiments for aptamer concentration were conducted using DPV at a concentration range of 0–5 µM, Fig. 8. The optimum concentration of 0.3 µM was obtained, since this concentration gives the lowest current. The increase after 0.3 µM could be due to the saturation. Electrode saturation is a known factor in electrochemical biosensors.^44^ Lower currents indicate better biomarker binding to the aptamer. All the DPV experiments were carried out in triplicates and averaged.

**Fig. 9:**
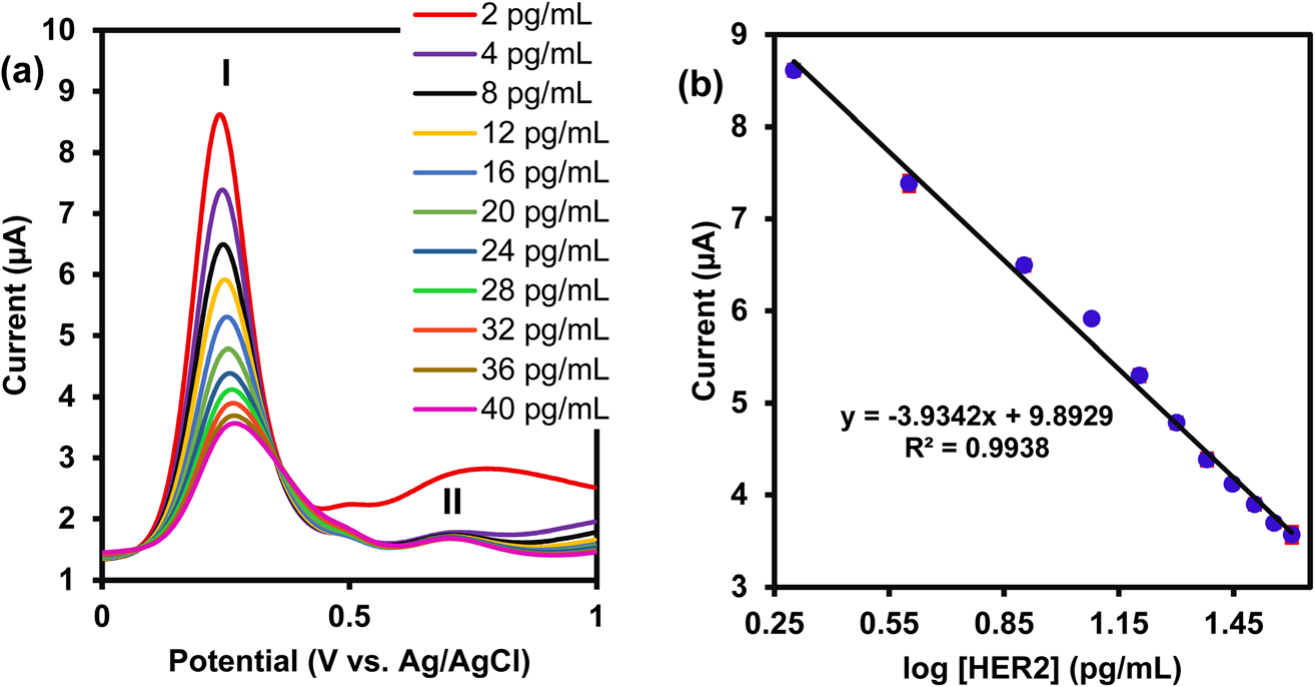
(a) DPV plots at various concentrations of HER2 (2.0–40 pg mL^−1^) in 1.0 mM [Fe(CN)_6_]^3−/4−^ containing 0.10 M KCl in 10 mM PBS (pH 7.4) and (b) calibration curve of current (µA) *versus* log[HER2] (pg mL^−1^) for GCE/Im-COF/Apt. Scan rate 10 mV s^−1^.

Using the optimized aptamer concentration of 0.3 µM, the bio-electrochemical interaction between HER2 and anti-HER2 aptamer modified electrodes (GCE/CoTAPc/Apt and GCE/Im-COF/Apt) were examined using DPV in 1 mM [Fe(CN)_6_]^3−/4−^ (in 0.1 M KCl) in 10 mM PBS (pH 7.4). The electrodes were incubated at various HER2 concentrations and current response of the ferri/ferrocyanide peak (I) was monitored. Fig. 9a and S5a illustrate DPV current responses against increasing HER2 concentrations. Inverse proportionality in electrochemical bio-detection between current response and analyte concentration is known.^45^ The gradual decrease in current response (peak I) as HER2 concentration increases is observed in this work (Fig. 9a and S5a). These observations arise from electrode inhibition by HER2 anti-HER2 aptamer bio-interactions. Electrode inhibition by increasing HER2 concentration results in poor electron transfer, hence the gradual decrease. The appearance of peaks (II) for both Fig. 9a and S5a are due to high sensitivity of DPV.^46^ In addition, Fig. S5a shows two redox peaks for II whereas CV (Fig. 6a) shows one for GCE/Im-COF/Apt only. The redox peaks (II) are due to Co(ii)/Co(iii) (+0.5 V) and Pc ring (+0.7 V) redox processes.^36,37^

The calibration curves shown in Fig. 9b and S5b were used to calculate limits of detection (LOD). Both electrodes show good correlation coefficients (*R*) of 0.994 (GCE/Im-COF/Apt) and 0.993 (GCE/CoAPc/Apt). To estimate the LODs, 3*δ*/*s* equation was employed, where *δ* is standard deviation of the blank (without HER2) and *s* is the slopes of the regression plots and sensitivity (Fig. 9b and S5b). The obtained values are summarized in Table 2. The π extension of phthalocyanine *via* covalent organic framework shows improved electrocatalytic properties, where GCE/Im-COF/Apt has the lowest LOD (0.093 pg mL^−1^) and higher sensitivity (3.93 µA pg^−1^ mL^−1^) when compared to GEC/CoTAPc/Apt (2.49 µA pg^−1^ mL^−1^) and LOD of 0.39 pg mL^−1^, Fig. 9b and S5b. Excellent electrocatalytic properties of the COF may also be attributed to high surface coverage. This facilitates easy and fast electron transfer. The obtained LODs are much lower than clinical standard of ∼15 ng mL^−1^. Our designed sensor is comparable to (and even better than) some reported sensors.^18,47–50^

**Table 2:** A summary of analytical parameters for the detection of HER2 (sensitivity and limit of detection (LoD)) measured in [Fe(CN)_6_]^3−/4−^ (1 mM) in 0.1 M KCl and 10 mM PBS (pH 7.4)^a^

Electrode	LCR (pg mL^−1^)	LOD (pg mL^−1^)	Detection technique	Ref.
GCE/CoTAPc/Apt	2–40	0.39	DPV	TW
GCE/Im-COF/Apt	2–40	0.093	DPV	TW
GCE/Fe_3_O_4_@TMU-21–MWCNT/Ab	1–1000	0.30	Amperometric	47
HRP–Ab–AuNPs@COF	0.5–1000	0.41	Chronocolorimetric	18
AuNPs@PDA@UiO-66	5–15000	5	Fluorescence	48
Antibody/N-CQDs/GS	100–1000	4.8	DPV	49
nanoD/AuNPs	1–50000	0.29	DPV	50

a LCR = linear concentration range, TW = this work, Ref. = references, MWCNT = multiwalled carbon nanotubes, Ab = antibody, AuNPs = gold nanoparticles, HRP = Horseradish peroxidase, PDA = polydopamine, N-CQDs = nitrogen-enhanced carbon quantum dots, GS = graphite sheet, nanoD = nanodiamonds. UiO-66 is a zirconium-based metal–organic framework (MOF) developed at the University of Oslo (UiO). TMU-21 is an imine-functionalized, 3D porous zinc(ii)-based metal–organic framework (MOF) developed at Tarbiat Modares University (TMU).

#### 3.3.1 Repeatability and stability studies

Accuracy in biosensors is important to assess possibilities for real and clinical applications. The repeatability studies were conducted in triplicates at 2 pg mL^−1^ to determine relative standard deviation (% RSD). The obtained values are shown in Table S1, where GCE/Im-COF/Apt obtained very low % RSD value of 0.21%.

**Fig. 10:**
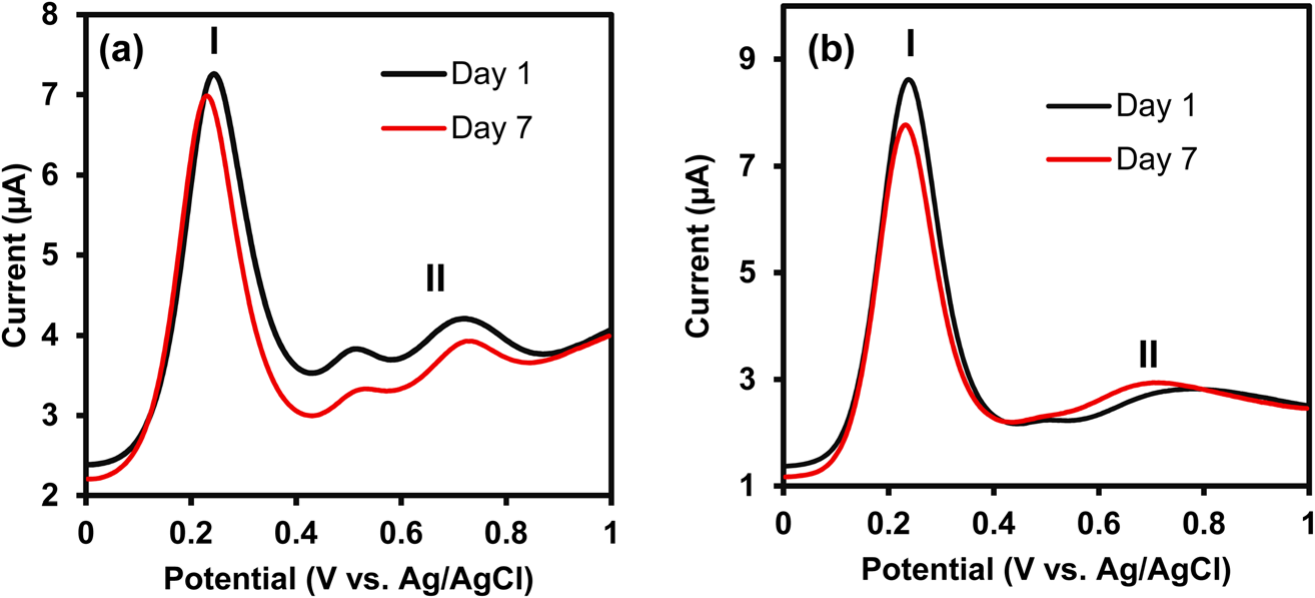
DPV scans for: (a) GEC/TAPc/Apt, (b) GCE/Im-COF/Apt stability studies at 2.0 pg per mL HER2 concentration in 1 mM [FeCN_6_]^3−/4−^ (in 0.1 M KCl) in 10 mM PBS.

The stability of the designed biosensors was assessed by storing the electrode at 4 °C for seven days. DPV scans were conducted for day 1 and after seven days in 1 mM [FeCN_6_]^3−/4−^ (in 0.1 M KCl) in 10 mM PBS. Changes in current response were observed and investigated by relative standard deviation between day one and seven (Fig. 10a and b). The calculated % RSD values show better stability for GCE/Im-COF (3.5%) when compared to GCE/CoTAPc/Apt (7.9%), Table S1. Further investigation on aptamer immobilisation stability was quantified by estimating % signal retention. The % retention is calculated using % retention = (*I*_*t*_/*I*_0_) × 100, where *I*_*t*_ is current response at specific time (day 7) and *I*_0_ is initial current response (day 1). Both our electrodes show excellent stability and signal retention ≥ 90% after seven days storage, Table S1.

#### 3.3.2 Selectivity studies

Selectivity experiments were conducted to further investigate the accuracy and sensitivity for our designed biosensor (GCE/Im-COF/Apt). Biological elements such as bovine human serum, cysteine and glucose were utilized. These biological elements are known to coexist with cancer biomarkers such as HER2, hence the use of mixed solution method.^51,52^ The current response was measured using DPV at ×10^3^ higher interferent concentrations than 2 pg mL^−1^ of HER2. Eqn (3) was employed to estimate the selectivity coefficients in the presence of interferents:^53^3
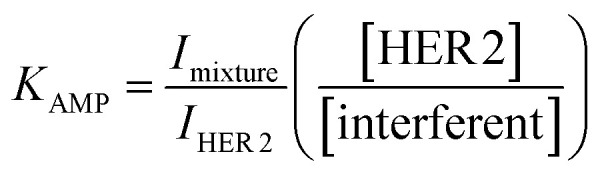
where *I*_mixture_ and *I*_[HER2]_ are DPV background corrected currents, [HER2] and [interferents] represents concentrations of HER2 and interferents, respectively. *K*_AMP_ is the selectivity coefficient.

The DPV scans in Fig. S6 show slight differences in peak heights and minimal effects on electrode's sensitivity. To further understand selectivity, selectivity coefficients were estimated from eqn (3). The calculated *K*_AMP_ values were 9.04 × 10^−4^ (bovine human serum), 1.10 × 10^−3^ (cysteine), 1.07 × 10^−3^ (glucose) and 8.80 × 10^−4^ (mixture). The values show selectivity coefficients closer and less than ×10^−3^, which is an indication of non-interfering species.^54^

#### 3.3.3 HER2 detection in human serum

The human serum analysis was conducted to assess our sensor performance for real-life clinical applications. The experiments were carried out in PBS pH 7.4 and diluted human serum. Human serum was diluted 500 times with PBS (1/500-fold) to overcome sample matrix.^55^ The resulting diluted human serum was further spiked with various HER2 concentrations (2, 4, 8 pg mL^−1^). Percentage recoveries were estimated to be 84.0%, 78.8%, and 103.0%, respectively (Table S2). The obtained results show satisfactory percentage recoveries for real-life application.

## 4. Conclusions

In this work, a novel imide-linked phthalocyanine based covalent organic framework (Im-COF) was successfully synthesized and utilized for electrode modification towards ultrasensitive electrochemical detection of the HER2 breast cancer biomarker. Comprehensive characterisation of the material confirmed the formation of stable imide linkages, pronounced ππ stacking within the 2D layered architecture, and significantly enhanced thermal robustness relative to the molecular precursor CoTAPc. The structural and electronic features collectively contributed to improved charge-transfer kinetics and electrochemical activity. The resulting GCE/Im-COF/Apt biosensor gave exceptional analytical performance with remarkably low detection limit of 0.093 pg mL^−1^. The sensor demonstrated excellent selectivity, stability and repeatability mainly due to advantages of combining highly ordered Im-COF with physisorbed anti-HER2 aptamer. Notably, this study presents the first application of a phthalocyanine-derived imide-linked COF for HER2 detection, highlighting its significant potential for point-of-care diagnostics and other clinically relevant biosensing applications.

## Author contributions

L. Ncwane: methodology, investigation, formal analysis, writing – original draft, data curation, conceptualization. P. Mashazi, T. Nyokong: writing – review and editing, validation, supervision.

## Conflicts of interest

There are no conflicts to declare.

## Supplementary Material

RA-OLF-D6RA04581B-s001

## Data Availability

Data will be made available upon request. Supplementary information (SI) is available. See DOI: https://doi.org/10.1039/d6ra04581b.
