# Supplementary material for: Imide-linked phthalocyanine-based covalent organic framework for electrochemical sensing of human epidermal growth factor receptor 2
Source: RSC Adv. 2026 Jul 7;16(36):38297–305. doi: 10.1039/d6ra04581b (PMC13338859; doi:10.1039/d6ra04581b)
Supplement: Supplementary file 1 [file RA-016-D6RA04581B-s001.pdf]

Supplementary Information (SI) for RSC Advances.
This journal is © The Royal Society of Chemistry 2026

Supporting Information

for

Imide linked phthalocyanine based covalent organic framework for electrochemical sensing of human epidermal growth factor receptor 2.

Lunathi Ncwane, Philani Mashazi, Tebello Nyokong

[a]Institute for Nanotechnology Innovation, Rhodes University, P. O Box 94, Makhanda, South Africa.

1. MATERIALS

Acetone, dimethylsulfoxide (DMSO) and dimethylformamide (DMF) were obtained from Merk. Pyromellitic dianhydride, N-methylpyrrolidone (NMP), pyridine, n-butanol, iron ferricyanide, and iron ferrocyanide, Na_2HPO_4, NaH_2PO_4 and human epidermal growth response factor 2 (HER) were purchased from Sigma-Aldrich. Phosphate buffer saline (PBS, pH 7.4) was prepared using appropriate amounts of Na_2HPO_4, NaH_2PO_4 and NaCl from Type II water which was obtained from an Elga PURELAB Chorus 2 (RO/DI) system. The primer 1 DNA aptamer (/5AmMC6/GG GCC GTC GAA CAC GAG CAT GGT GCG TGG ACC TAG GAT GAC CTG AGT ACT GTC C) (31.6 nmol, >90% purity) was purchased from Integrated DNA technologies (IDT), South Africa.

2. EQUIPMENT

UV–visible spectra data was obtained from Shimadzu UV–Vis 2550 spectrophotometer in the range of 300–800 nm. X-ray powder diffraction (XRD) patterns were recorded on a Bruker D8 Discover equipped with a Lynx-Eye Detector,

using CuKα radiation (= 1.5405 Å, nickel filter). Thermogravimetric analysis (TGA) was conducted using a Perkin-Elmer TGA 7 analyzer, at a heating rate of 10 °C min^{-1} under nitrogen gas from 50 °C up to 900 °C. Infrared spectroscopy was performed using a Bruker Alpha IR (100 FT-IR) spectrophotometer. Atomic force microscopy (AFM) images were recorded using the non-contact mode in air with MFP-3D Origin at a scan rate of 0.50 Hz. X-ray photoelectron spectroscopy was done with a Kratos Axis Ultra DLD, using an Al (monochromatic) anode, equipped with charge neutralizer and the operating pressure kept below 5×10^{-9} torr. Curve fitting was performed using a Gaussian-Lorentzian peak shape after performing a linear background correction using Vision 2 software.

Cyclic voltammetry (CV) (potential window of -0.5 to 1.0 V vs Ag/AgCl for ferri/ferrocyanide) experiments were performed using Autolab Potentiostat PGSTAT 302 electrochemical workstation (driven by GPES software version 4.9). Electrochemical impedance spectroscopy (EIS) studies were performed at 0.10 Hz to 100 kHz frequency range using a sinus amplitude of 10 mV and an applied potential of E_p = 0.24 V for $[Fe(CN)_6]^{3-/4-}$, with the DC application time of 5 s, using an Autolab Potentiostat PGSTAT30 equipped with Nova software version 2.1. A non-linear least squares (NLLS) method based on the EQUIVCRT programme was used for automatic fitting of the obtained EIS data. Differential pulse voltammetry (DPV) was performed at a potential window of 0 V to 1 V with pulse amplitude of 0.025 V and a step width of 0.05 s, using Autolab Potentiostat PGSTAT 302 electrochemical workstation (driven by GPES software version 4.9).

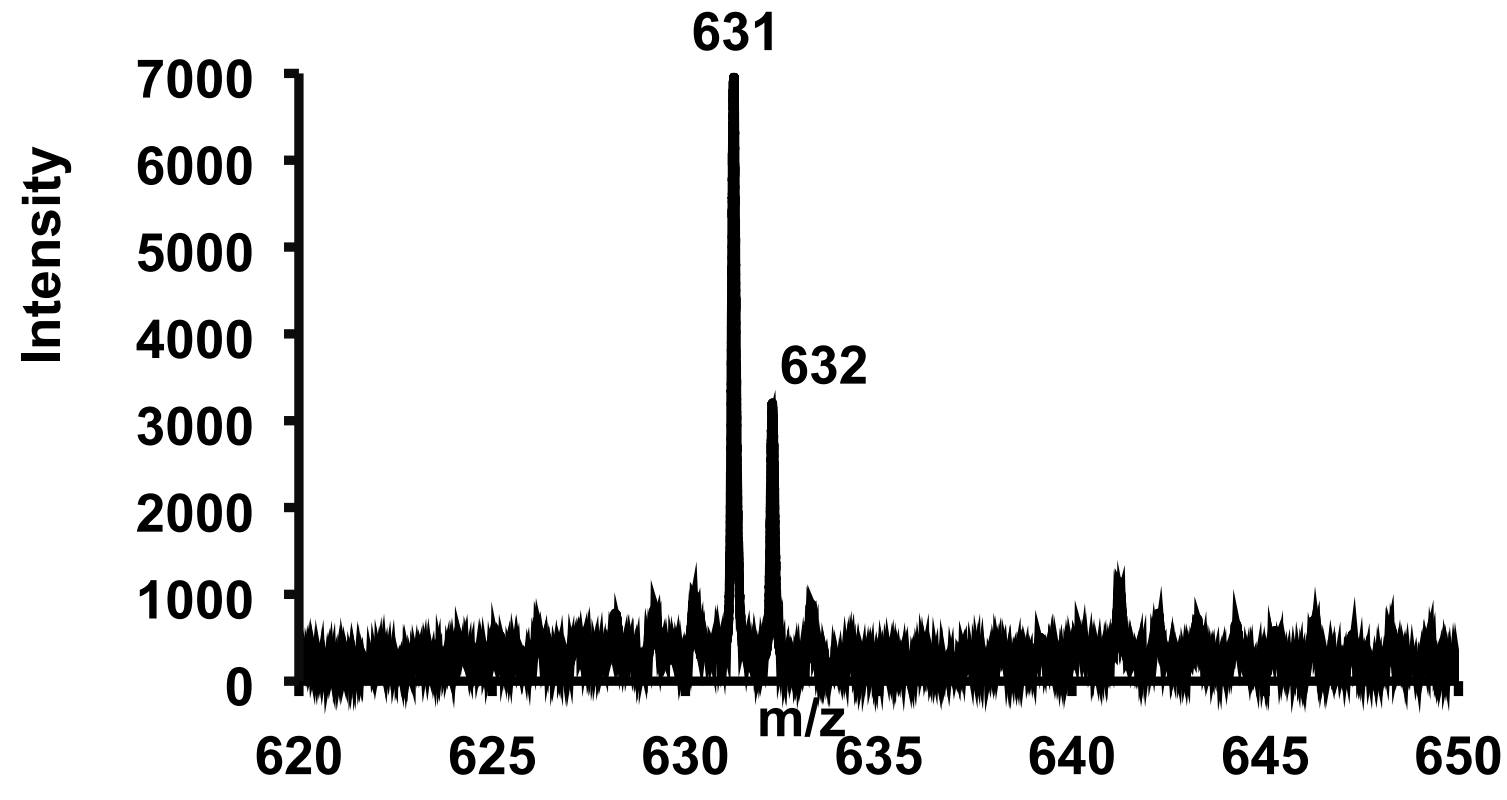

Figure S1: Time-of-Flight Secondary Ion Mass Spectrometry (TOF-SIMS) spectrum of CoTAPc.

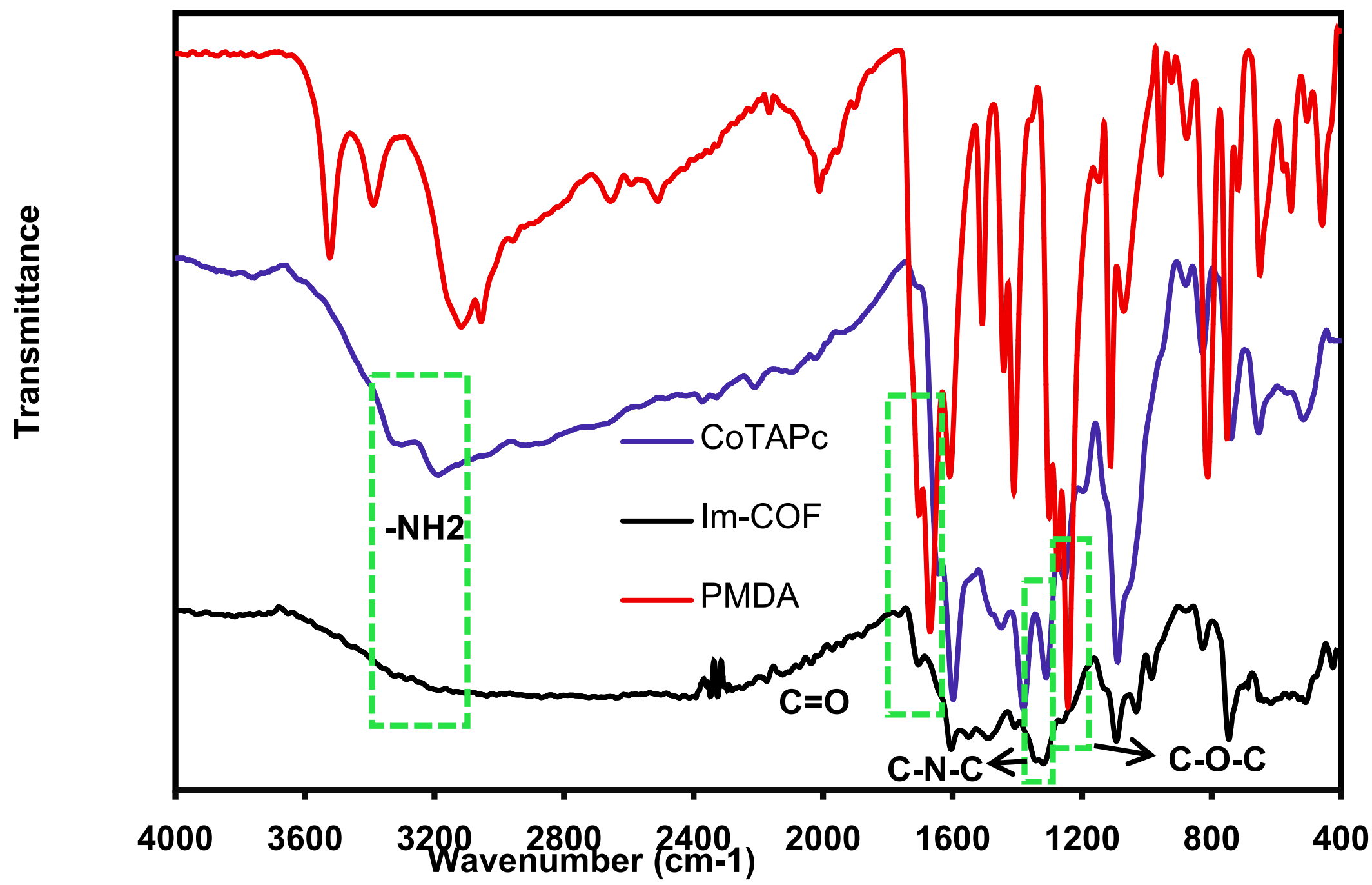

Figure S2: IR spectra of pyromellitic anhydride (PMDA), CoTAPc and Im-COF.

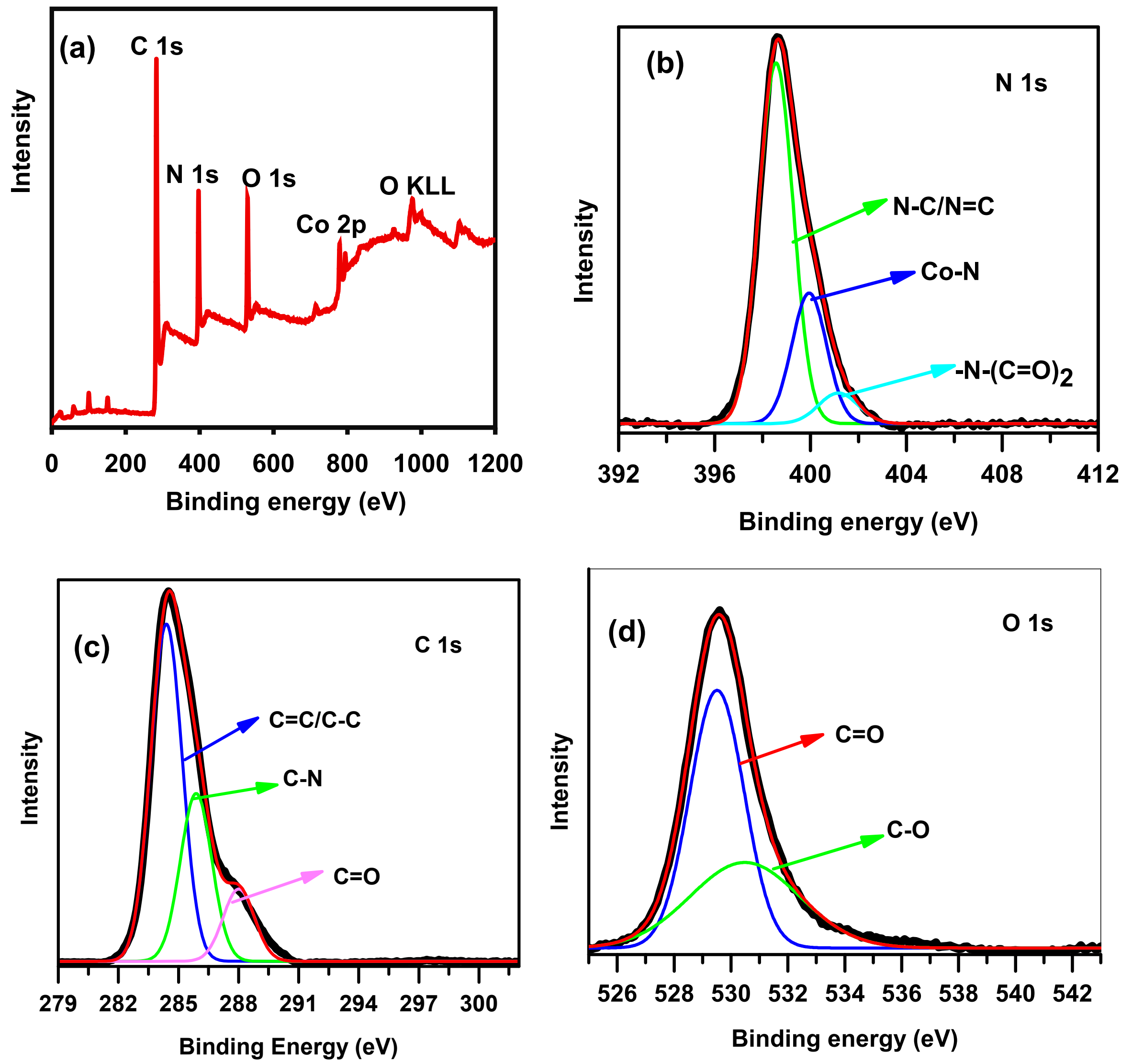

Figure S3: XPS spectra of the Im-COF: **(a)** Survey scan, and high-resolution spectra of **(b)** N 1s, **(c)** C 1s and **(d)** O 1s.

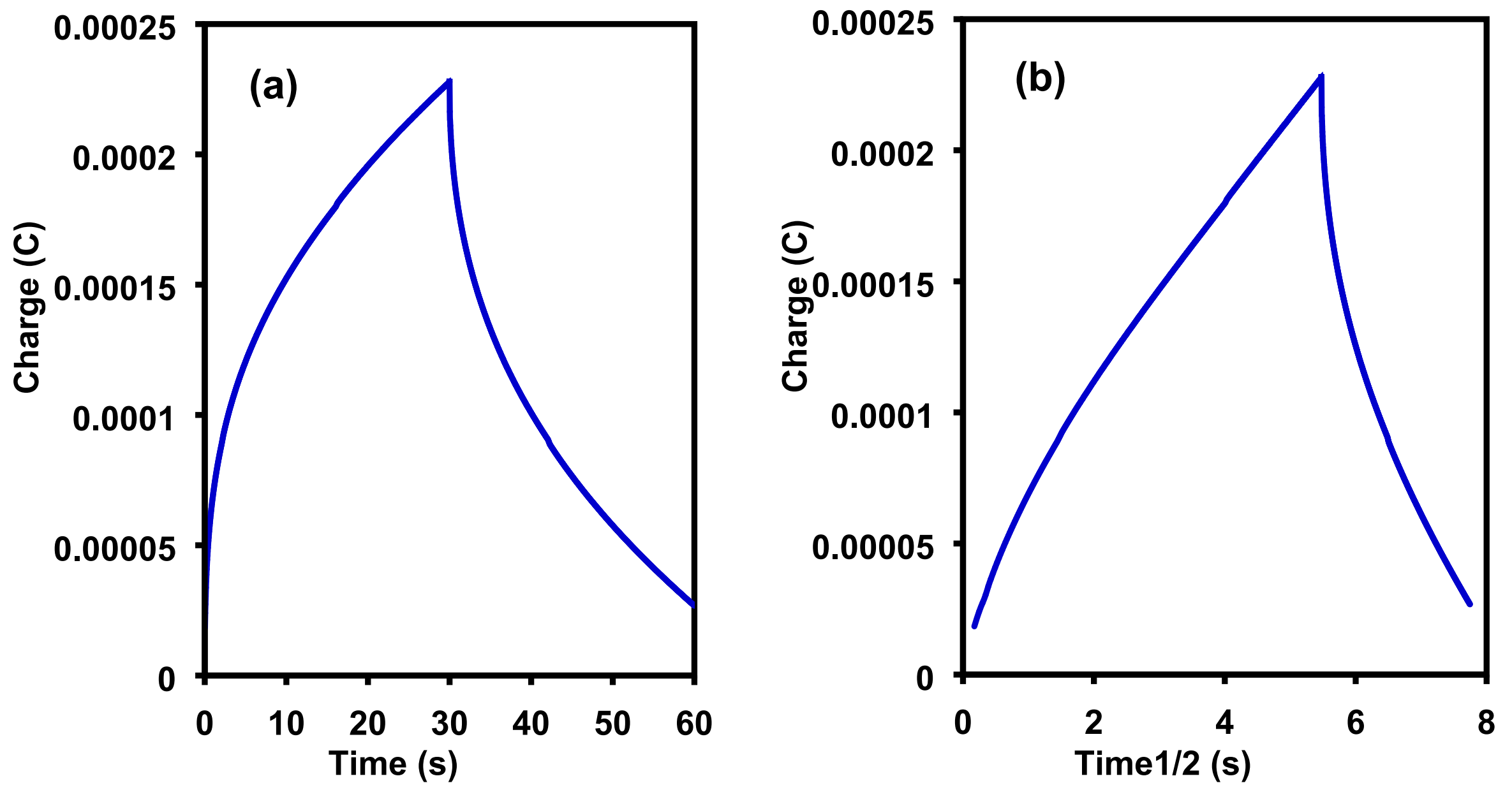

Figure S4: Chronocoulorimetry plots of GCE/Im-COF in 1 mM $[FeCN_6]^{3-/4-}$ (in 0.1 M KCl) in 10 mM PBS **(a)** time versus charge and **(b)** square root of time versus charge.

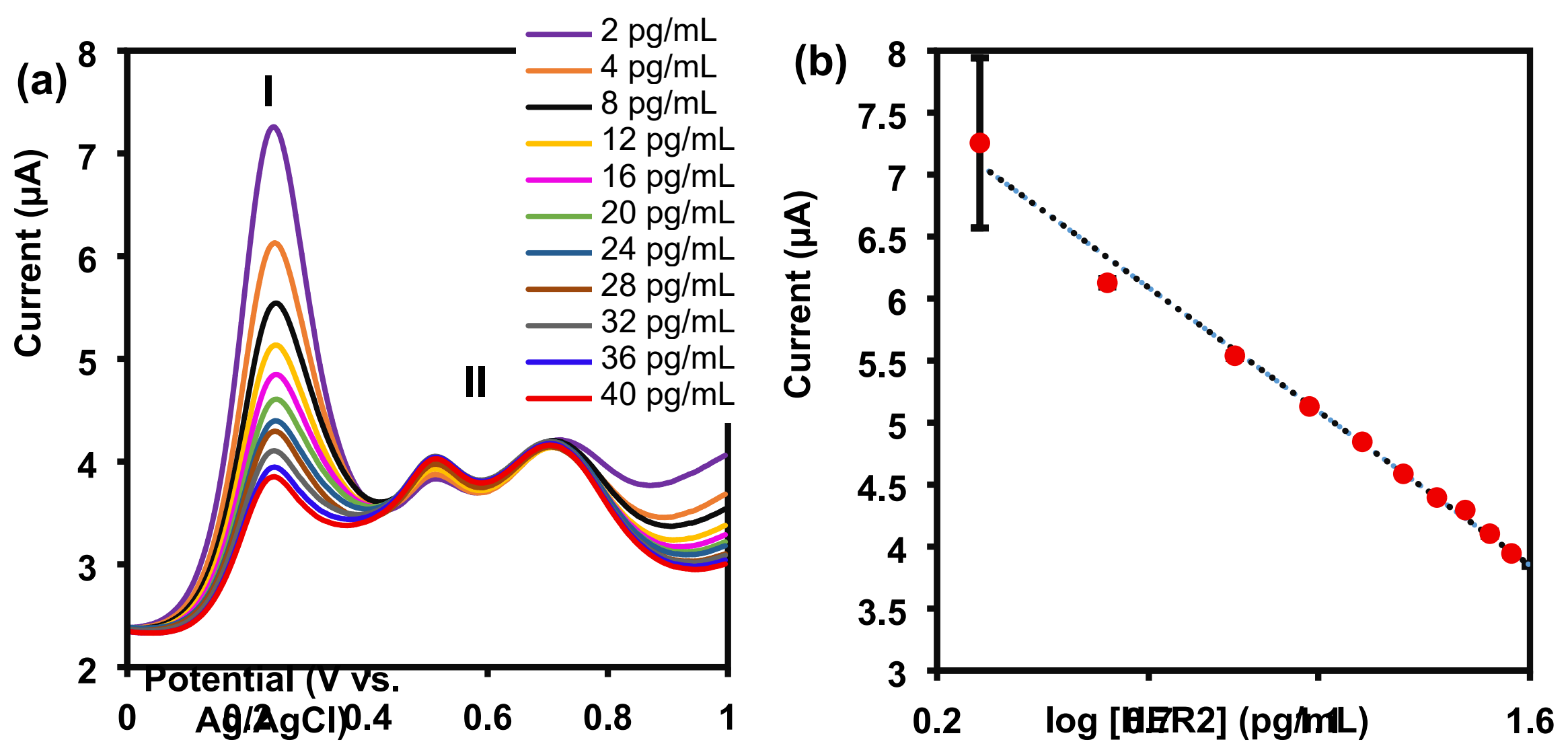

Figure S5: (a) DPV plots at various concentrations of HER2 (2.0 – 40 pg/mL) in 1.0 mM $[Fe(CN)_6]^{3-/4-}$ containing 0.10 M KCl in 10 mM PBS (pH 7.4) and **(b)** Calibration curve of current (µA) versus log [HER2] (pg/mL) for GCE/CoTAPc/Apt.

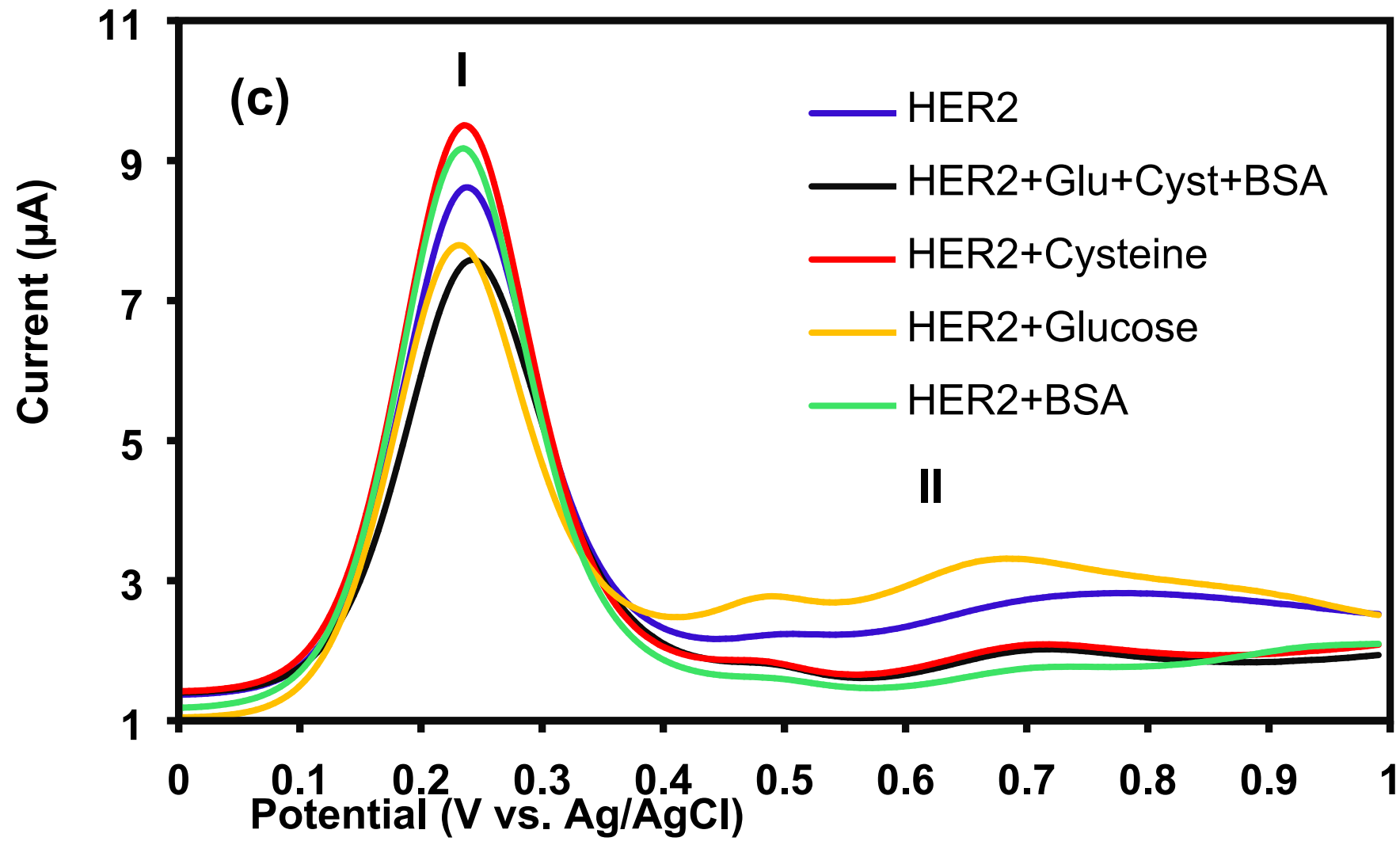

Figure S6: DPV scans for selectivity studies of GCE/Im-COF/apt at 2.0 pg/mL HER2 concentration in 1 mM $[FeCN_6]^{3-/4-}$ (in 0.1 M KCl) in 10 mM PBS.

Table S1: Percentage relative standard deviation values (%RSD) for stability, repeatability and % signal retention of the aptasensors for the detection of 2.0 pg/ml HER2.

Electrodes	Stability (%RSD)	% Retention	Repeatability (%RSD)
GCE/TAPc/Apt	7.9	96.2	9.4
GCE/Im-COF/Apt	3.5	90.2	0.21

Table S2: Summary of % recoveries for GCE/Im-COF/Apt

PBS		Human Serum		% [HER2] recovery
[HER2] (pg/mL)	Current (μA)	[HER2] (pg/mL)	Current (μA)	
2	3.06	1.68	2.90	84.0
4	2.93	3.15	2.81	78.8
8	2.79	8.24	2.67	103
